# The microbiota of the mother at birth and its influence on the emerging infant oral microbiota from birth to 1 year of age: a cohort study

**DOI:** 10.1080/20002297.2019.1599652

**Published:** 2019-04-26

**Authors:** Eimear Hurley, David Mullins, Maurice P. Barrett, Carol Anne O’Shea, Martin Kinirons, C. Anthony Ryan, Catherine Stanton, Helen Whelton, Hugh M. B. Harris, Paul W. O’Toole

**Affiliations:** a School of Microbiology, University College Cork, Cork, Ireland; b Cork University Dental School & Hospital, Cork University Hospital, Wilton, Cork, Ireland; c APC Microbiome Ireland, University College Cork, Cork, Ireland; d Department of Neonatology, Cork University Maternity Hospital, Wilton, Cork, Ireland; e Teagasc Research Centre Teagasc,, Cork, Fermoy, Ireland; f College of Medicine and Health, UCC, Cork, Ireland

**Keywords:** Microbiota, vagina, skin, mother, transmission, oral cavity, saliva, newborn, infant

## Abstract

**Background: **The acquisition of microbial communities and the influence of delivery mode on the oral microbiota of the newborn infant remains poorly characterised. **Methods:** A cohort of pregnant women were enrolled in the study (*n* = 84). All infants were born full term, by Spontaneous vaginal delivery (SVD) or by Caesarean section (CS). At delivery a saliva sample along with a vaginal/skin sample from the mother. Saliva samples were the taken from the infant within one week of birth, and at week 4, week 8, 6 months and 1 year of age. We used high-throughput sequencing of V4-V5 region 16S rRNA amplicons to compare the microbiota of all samples. **Results: **The vaginal microbiota had a lower alpha diversity than the skin microbiota of the mother, while the infant oral microbiota diversity remained relatively stable from birth to 8 weeks of age. The oral microbiota of the neonate differed by birth modality up to 1 week of age (*p* < 0.05), but birth modality did not have any influence on the infant oral microbiota beyond this age. **Conclusions: **We conclude thatbirth mode does not have an effect on the infant oral microbiota beyond 4 weeks of age, and the oral microbiota of infants continues to develop until 1 year of age.

The human microbiome is made up of a rich environment of microbes and has been described as ‘a forgotten organ’ [1]. These microbes play an important role in modifying human health and disease [2–4]. Advances in molecular methods analysing the microbiome have allowed us to study various body sites in great depth, with the most extensively studied microbiota site in infants being the gut microbiota [5–7]. As the infant grows, the gut microbiota develops and matures from birth until 2–3 years of age, to establish a more adult-like ‘stable’ microbiota compositional state [8,9]. In recent years, there has been a surge of interest studying the effect of birth mode and feeding on the infant microbiota from birth until 2–3 years of age, to gain an understanding of the importance this period plays in programming the microbiota and innate immune system for later in life. While the establishment of the gut microbiota composition has been demonstrated to be affected by birth mode, cessation of breastfeeding and weaning onto solids [5–7,10–14], there are fewer studies demonstrating these effects on the oral microbiome of the infant [15–17].

The initial microbial exchanges between mother and infant at birth and shortly thereafter are fundamental to the infant microbiota, as these early colonisers play a very important role in the development of the neonate’s immune system and long term in the activity and function of the microbiota [18,19, 20]. During vaginal delivery, the newborn infant is colonised by the mothers’ vaginal fluid microbiota and intestinal microbiota, whereas the microbiota of those infants born via caesarean section (CS) share a close resemblance to the skin microbiota of the mother [11,15,21,22]. CS delivery has been shown to have an effect on the microbiota composition, and this change is associated with medical complications, such as coeliac disease, asthma, autism and obesity [23–27]. CS delivery is often medically necessary due to maternal or foetal complications and can be accompanied by antibiotic administration before and after birth, which can also impact the gut microbiota [1,13,28], with little impact on the oral microbiota [29]. While the CS rates worldwide are increasing [30] and because of the increased risk of complications associated with CS, there is an increased focus investigating the effect birth mode has on the newborn microbiota with studies exploring the possibility of recolonising the newborn born by CS, with swabs of the mothers vaginal fluid, in an effort to mitigate any disruption in the loss of these important early microbes [31].

Birth mode is known to influence the oral microbiota in infants at 3 months of age [16] and infants born by vaginal delivery have a higher oral microbial diversity than CS infants in the first 6 months post-birth [17]. A higher abundance of health-associated streptococci and lactobacilli were detected in the oral cavity of vaginally delivered infants [32], while infants delivered by CS acquired *Streptococcus mutans* nearly 12 months earlier than vaginally delivered infants [33]. The influence of birth mode on the oral microbial composition of infants has been demonstrated; however, saliva samples were taken from infants after birth at a mean age of 8.25 months [34], 3 months [16] and at 1, 3 and 6 months [17] without taking vaginal or skin samples from the mother, based on the mode of delivery. There are some limitations in these studies, particularly with sampling of the infant at only one time point [16,32], while the strength of the study by Boustedt et al. [17] was increased by longitudinal sampling within 2 days of birth to 6 months, which allowed examination of change in the oral microbiota over time. Studies of the impact of the feeding modality (breast or formula) on the oral microbiome have also demonstrated microbiota compositional changes [35, 36]. *Lactobacillus* species, such as *Lactobacillus grasseri*, were detected at higher abundance in breastfed infants, compared to formula-fed infants [37], and these oral lactobacilli have antimicrobial properties and probiotic qualities [36].

Despite the increased work studying the oral microbiome of children and adults [38–41], there is a need for more in-depth research to investigate the oral microbiota from birth of the edentulous neonate, where to our knowledge there are only a few studies using high-throughput sequencing to examine the neonatal oral microbiota [15,42,43]. In addition to this, the use of high-throughput sequencing is needed to examine the oral microbiota by longitudinal sampling of the neonate from birth through to older age, taking into account factors, such as eruption of teeth, feeding modality at birth (breast or formula) and introduction of solid food. Understanding the oral microbiota at birth, prior to eruption of teeth and longitudinal sampling, may provide us with vital information on the evolution of the oral microbiome, birth mode influence and potential early indicators of disease risk (caries) in these infants. The main hypotheses to be tested in this study were therefore that there was a difference in the composition of the oral microbiome of children (a) who are delivered by CS or spontaneous vaginal delivery (SVD) and (b) from birth to 1 year of age.

In this prospective study, we employed 16S rRNA gene amplicon Illumina sequencing to investigate the microbiota of the mother across different body sites at birth (vagina/skin and oral cavity) and the oral microbiota of their infants at birth forming mother–infant pairs, followed by longitudinal sampling at five time points until 1 year of age (1 week, 4 weeks, 8 weeks, 6 months/24 weeks and 1 year of age). All infants were from a single geographical area (Cork, Ireland), full-term delivered, initially breastfed for 4 weeks minimum and born by different birth modes (SVD or CS delivery). The main objectives of this study were (1) to describe and compare the microbiota of the different body sites of the mother (vagina, skin, oral cavity) with and that of their infant (oral cavity), (2) to identify if there is any influence on mode of delivery on the oral microbiota of the infant, (3) to identify if breastfeeding and the length of breastfeeding has an impact on the oral microbiota of the infant and, finally, (4) to describe the oral microbiota changes over time as the infant develops and grows from birth to 1 year of age (1 week, 4 weeks, 8 weeks, 6 months/24 weeks and 1 year of age).

## Materials and methods

### Study design, ethics and recruitment

The study design was to recruit a cohort of mother–infant dyads for longitudinal sampling (from birth to 1 year of age). This cohort was given the title ORALMET, with ‘oral’ standing for the oral cavity and ‘met’ standing for metagenomics. The study cohort included pregnant mothers recruited antenatally, and their infants whom where followed and sampled from birth to 1 year of age. Mothers were approached and recruited antenatally between February 2012 and May 2014 at the Cork University Maternity Hospital (CUMH). Ethical approval was obtained from the Cork Teaching Hospitals Clinical Research Ethics Committee (reference code: ECM 3 (cc) 01/07/14). All mothers were consented for the study within 1–2 months prior to their estimated date of delivery, and two groups of mother–infants dyads (*n* = 185) were created based on their mode of delivery (full-term SVD infants and full-term CS delivered infants). True definition of a newborn baby aged 28 days or less is defined as a ‘neonate’ and aged 29 days or greater is defined as an ‘infant’ [43], but in this study, although sampling began at birth, all neonates and infants are defined under the same label ‘infant’. Body sites included in sampling were vagina, skin, oral cavity of the mother and oral cavity of the infant. All SVD and CS deliveries were uncomplicated, and all infants were born full term. Inclusion criteria, applied to both SVD and CS delivered infants, were that all infants were born full term (>35 weeks’ gestation) and medically healthy. Neonates did not receive antibiotic treatment at birth and infants were breastfed for a minimum of 4 weeks post-partum. Mothers who delivered neonates by CS were all given IV antibiotics prior to the surgery. If antibiotics were administered to the infant during the first year of life, this was recorded along with the duration of breastfeeding. Bioinformatic analysis was not performed as the infant numbers with antibiotic intake were low and previous findings demonstrated little impact on the salivary microbiota by antibiotic intake (Zaura et al., 2015). Exclusion criteria included breastfeeding duration shorter than 4 weeks, formula feeding and geographical area outside the practical catchment area. The recruitment resulted in the enrolment of a final number of 84 mothers into the study. We did not collect all oral samples from all infants and did not have consistency of collection across all the time points; the main reasons for loss of collection were introduction of formula feeding, inability to contact the mother to collect the sample and families’ moved location. Skin samples from 38 mothers of CS infants were collected, and 37 vaginal samples from SVD delivered infants (Supplementary Table 1). Some loss of samples from the mothers’ skin or vaginal samples were not collected at birth due to circumstances, such as emergency nature of the birth and missing the sampling opportunity. We were also short of two saliva samples from the mother due to circumstances at the time of birth which did not allow us to timely collect this sample. Information about the infants was collected at delivery using medical records at CUMH, and further data were collected from each mother–infant pair, when the infant was 1 year old.

### Sample collection

A CatchAll™ collection swab (Cambio, UK), with a hard pack for storage after collection, was used to collect all samples from each of the following body sites: vagina, skin and oral cavity (saliva) [15,44]. From the mother, a saliva sample was collected and a skin or vaginal sample depending on CS or vaginal delivery, respectively. For the SVD-delivered infants, using a CatchAll™ collection swab, the midwife or gynaecologist collected mid-vaginal samples from the mother within 1 h before delivery. For CS-delivered infants, the mother’s skin (right and left forearm area) was swabbed after moistening a CatchAll™ collection swab in sterile water, within 1 h before delivery. For emergency CS deliveries, the skin swabs were taken within 1 h after delivery. The vaginal and skin samples were placed immediately on dry ice and transported to the laboratory, where they were frozen until further analysis and stored at −80°C. Saliva samples from the mother were collected using a CatchAll™ collection swab, from pooled saliva within the floor of the oral cavity within 1 h of the infant being delivered. The saliva sample from the mother was labelled as ‘Saliva’ in the “Results” section. A CatchAll™ collection swab was used to collect pooled unstimulated saliva in the floor of the mouth for 1–2 min. For the infants, a saliva sample was collected using a CatchAll™ collection swab. The saliva sample collected from the infant’s oral cavity at each time point was labelled as ‘Oral week 1, Oral week 4, etc.’ in the “Results” section. The first saliva sample was collected from the newborn infants within 2 days of delivery, before the mothers left hospital (*n* = 77) (labelled as week 1). This was repeated again at 4 weeks (*n* = 61), 8 weeks (*n* = 60), 24 weeks (6 months) (*n* = 64) and at 1 year (*n* = 84) of age (Supplementary Tables 1 and 2). All saliva samples were placed immediately on dry ice, transported to the laboratory, where they were frozen until further analysis, and stored at −80°C.

### Sample extraction and processing

Extraction of DNA from all samples (vagina, skin and saliva/oral cavity) was carried out using the MO BIO PowerSoil DNA Isolation kit (Qiagen) along with the MO BIO PowerLyzer® 24 homogeniser with some initial optimisation for extraction from using a CatchAll™ collection swab rather than a soil sample as previously described [15,44,45]. The sample (vagina/skin/saliva) was contained in a CatchAll™ collection swab on the end of a collection tube, which was thawed before processing. The tube was cut 1 cm above this swab, and this was inserted into the PowerBead tubes, to which 60 µl of solution C1 had been added. Tubes were incubated at 65°C for 10 min and then shaken horizontally at maximum speed for 2 min, using the MO BIO vortex adapter. The remainder of the protocol was followed as per manufacturer’s instructions. DNA was visualised on a 0.8% agarose gel and quantified using the Nanodrop 1000 (Thermo Scientific, Ireland). DNA was then stored at −80°C.

### 16S rRNA gene amplification primers

Primers used for PCR amplification were the V4–V5 region primers 520F (AYTGGGYDTAAAGNG) and 926R (CCGTCAATTYYTTTRAGTTT) (see Supplementary Figure 1) due to their high classification accuracy and consistent results [46–48]. Primers for Illumina sequencing contain the sequencing primer-binding sites, forward or reverse 16S rRNA gene-specific primer and a 10-nt in-line multiplexing identifier (MID). Dual separate MIDs were attached to both ends of the PCR product (see Supplementary Figure 1) [5]. The V4–V5 amplicons for Illumina sequencing were generated using a two-step amplification procedure. The first step reaction mix contained 50 μl BIO-X-ACT™ Short Mix (BIOLINE), 10 μl of 2 nM forward and reverse primers, 50 ng genomic DNA and ddH_2_O to give a final volume of 100 μl. Cycling conditions were an initial 95°C, 5-min denaturation step; 30 cycles of 95°C for 15 s, 42°C for 15 s and 72°C for 30 s; and a final 10-min extension at 72°C. The products were purified using solid phase reversible immobilisation (SPRI) select beads (Beckman Coulter, IN) as per manufacturer’s instructions, using a 0.9:1 volume ratio of beads to product. The purified PCR products were eluted in 40 μl of ddH_2_O. DNA quantity was assessed via Quant-iT™ PicoGreen® dsDNA Assay Kit (Invitrogen™). The samples were pooled in equimolar amounts (20 ng per sample) and then sequenced by Eurofins Genomics (Eurofins Genetic Services Ltd., I54 Business Park, Valiant way Wolverhampton WV9 5GB, UK) using Illumina MiSeq 2 × 300 bp paired end technology. Nextflex Rapid library preparation was carried out by the company to attach bridge adaptors necessary for clustering. Sequencing of 16S DNA was carried out on the V4/V5 region using a Miseq (300 bp paired-end reads). Sequence data were stored on a Linux server and backed up on external hard drives.

## Bioinformatic analysis

### Sequence processing, OTU clustering and taxonomy assignment

The software FLASH (v1.2.8) was used to join paired-end reads. Paired-end reads with more than 25% incorrect bases in their region of overlap were excluded from subsequent steps. Qiime (v1.9.1) was used to extract barcodes (extract_barcodes.py) and for demultiplexing (split_libraries_fastq.py). Barcode bias was excluded by grouping all samples according to their forward and reverse barcodes (separately) and plotting those using principal coordinate analysis (PCoA) analyses to observe any separation of samples based on barcode (see Supplementary Figure 2). A permutational multivariate analysis of variance (PerMANOVA) test was used to check for significance (adonis) function in R from the vegan package.

The USEARCH (v8.0.1623) pipeline was used for the following steps: de-replication of reads (identical reads are represented by a single sequence), exclusion of reads shorter than 350 bp and longer than 370 bp, exclusion of unique reads, chimera filtering, Operation Taxonomic Unit (OTU) clustering at 97% identity and calculation of representative OTU sequences. Using USEARCH, all reads (including unique reads) were then mapped back to the representative OTU sequences to give the final OTU read count for each sample. The software fastQC (v0.11.3) was used after each filtering step to assess read quality. The sample number after sequence processing was 503

Part of the mothur (v1.36.1) [49] pipeline was used to run the Ribosomal Database Project (RDP) classifier using a filtered version of the RDP database in order to assign taxonomy down to genus level. The software SPINGO (v1.3) [50] was used to assign taxonomy at species level. For both mothur/RDP and SPINGO, confidence cut-offs of 80% were used [80% of kmers (nucleotide sequences of a given length) match those in species to which it is assigned].

### Alpha- and beta-diversity analysis

Alpha- and beta-diversity metrics were calculated in Qiime (v1.9.1) [51]. To calculate diversity metrics, several additional steps were carried out (also in Qiime). The OTU table was rarefied (single_rarefaction.py) at 10,540 reads (the lowest read count in the data set). Representative OTU sequences were aligned using pyNAST (align_seqs.py) and filtered to remove columns that do not contribute to phylogenetic signal (filter_alignment.py). A phylogenetic tree was generated using FastTree (make_phylogeny.py). This tree is necessary for phylogenetic alpha- and beta-diversity metrics. The rarefied OTU table was used in the calculation of all diversity metrics.

The following alpha-diversity metrics were calculated: chao1, Shannon (Shannon’s index), Simpson (Simpson’s index), observed species (OTU count) and phylogenetic (PD whole tree). The following beta-diversity metrics were calculated: weighted and unweighted UniFrac distances and Bray–Curtis dissimilarity.

### Statistics and data visualisation

All statistics and data visualisation were carried out in R (v3.2.3 and v3.4.0) (Statistical and Computing, Vienna 2016 [52]). Alpha-diversity box plots were created using the package ggplot2. Unpaired analysis was also completed, with the Mann–Whitney *U* test being used to compare two groups and Kruskal–Wallis for three or more groups, both by paired and unpaired analysis. A Mann–Whitney *U* test was used to test whether SVD- and CS-born babies differed significantly for each time point. A Kruskal–Wallis test was performed on alpha-diversity metrics for each infant time point, followed by Mann–Whitney *U* pairwise comparisons corrected using the Benjamini–Hochberg method [52]. Dunn test was performed for the pairwise comparison of the alpha diversity for the mother samples with the infant time points. PCoA plots of beta-diversity metrics were created using the package ade4. Statistical differences in beta diversity were tested using the adonis function from the vegan package. Taxon abundance bar plots were created using the packages reshape2, ggplot2 and ggthemes. Taxon abundance was normalised to sample proportions for the bar plots. Kruskal–Wallis tests and Benjamini–Hochberg pairwise tests were used to test for statistical difference in particular taxa at different time points. Clustering of mother–infant pairs based on beta diversity was performed using the hclust function from base R, and the plot created using the rafalib package. The heatmaps to investigate clustering of mother–infant pairs and infant time points based on genera abundances were created using the metagenomeSeq package in R.

## Results

### Birth mode does not drive separation of the oral microbiota of infants, while the oral microbiota of the infant clusters, from birth to week 8 of age, is independent of birth modality

**Figure 1.**
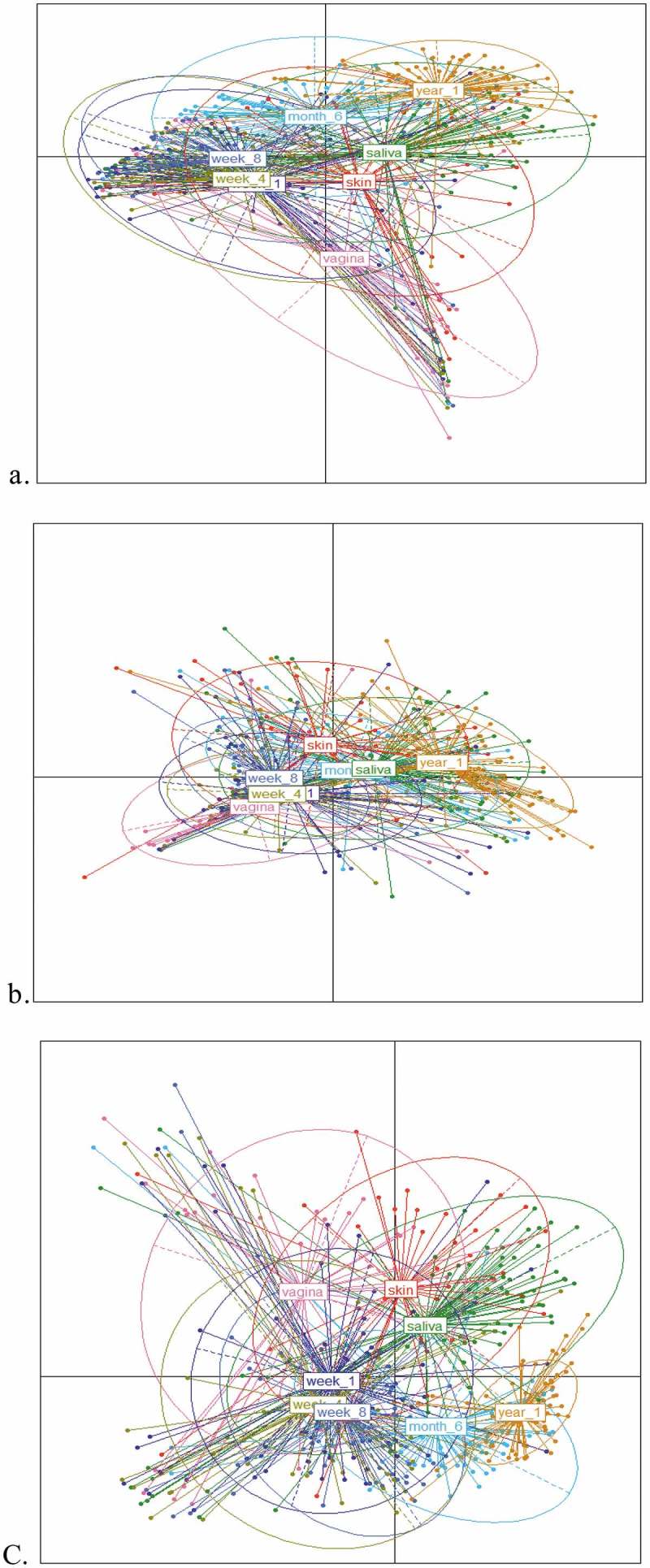
Principle coordinates analyses (PCoAs) on Spearman distance matrices of samples from mother (saliva, skin and vagina) and from infant oral cavity at 5 time points (weeks 1, 4, 8, 6 months and 1 year). (a) Bray–Curtis dissimilarity, (b) UniFrac weighted, and (c) unweighted UniFrac between the 8 groups (mother saliva/oral cavity, skin and vaginal and infant oral cavity at weeks 1, 4, 8, 6 months and 1 year). (a) Plot of principle coordinates using Bray–Curtis dissimilarity. Points are coloured according to group and ellipses describe the distribution of points for each group. Percentage variation explained: PCA 1 (18.5%) and PCA 2 (9.2%). (b) Plot of principle coordinates using weighted UniFrac distance. Points are coloured according to group and ellipses describe the distribution of points for each group. Percentage variation explained: PCA 1 (39.2%) and PCA 2 (11%). (c) Plot of principle coordinates using unweighted UniFrac distance. Points are coloured according to group and ellipses describe the distribution of points for each group. Percentage variation explained: PCA 1 (18.4%) and PCA 2 (6.7%).

To investigate the relatedness of the microbiome composition between samples including the maternal vagina, skin and their oral cavity (saliva) to their infants from week 1 to 1 year of age (at the following time points: 1 week, 4 weeks, 8 weeks, 6 months and 1 year), we generated PCoA (principle coordinate) plots showing relatedness by two established metrics, Bray–Curtis dissimilarity and UniFrac distances. The Bray–Curtis plot (see Figure 1(a)), weighted UniFrac (see Figure 1(b)) and unweighted UniFrac (see Figure 1(c)) both demonstrate substantial overlap between each group, indicating similarity in the general composition of the microbial taxa. However, using PerMANOVA to compare the microbiota of the mother (vagina, skin and oral cavity), with the oral microbiota of the infants at different time points, we found statistically significant differences between each group, as measured by each metric (*p* < 0.001) (Table 1).

**Table 1.** Significance was calculated using permutational multivariate analysis of variance (PerMANOVA).

	Axis 1	Axis 2	PerMANOVA (*p*-value)
Bray–Curtis	18.5	9.2	<0.001***
Weighted UniFrac	39.2	11	<0.001***
Unweighted	18.4	6.7	<0.001***

*p < 0.05; **p < 0.01; ***p < 0.001.

The Bray–Curtis (see Figure 1(a)) and weighted UniFrac (see Figure 1(b)) demonstrate overlap between the earlier ages of the infant (week 1, week 4 and week 8) along with the vagina of the mother in the weighted PCoA. Weighted UniFrac is sensitive to the differences in the presence/absence and abundance/proportions of OTUs. There is evidence of tight clustering of weeks 1, 4 and 8 infant oral microbiota samples along with less clustering together with 6 months and 1 year samples.

Plotting data sets, using the second UniFrac metric and unweighted UniFrac distances (see Figure 1(c)), illustrate tight clustering again of the early gestation age time points of the oral microbiota of the infant (week 1, week 4 and week 8), with less overlap with the increased age (6 months and 1 year). This index measures the presence and absence of taxa only and does not take taxon abundance into account. The clustering and overlap of the infant oral microbiota at 1, 4 and 8 weeks of age indicate sharing of rare taxa at this early age, while the evidence of minimal overlap of the infant oral microbiota at 1 year of age indicates less sharing of taxa at this age and demonstrates that the infant oral microbiota from birth to 1 year of age is continuously developing and changing. This is further supported by the oral microbiota of infants at 6 months, positioned between weeks 1–8 and year 1, thus highlighting a gradual change in the microbial composition from week 8 to 6 months and finally to 1 year of age, where the microbiota appears more unique in its composition (see Figure 1(c)).

### The diversity of the oral microbiota of the infant is influenced by birth mode at 1 week of age, but not beyond 1 week of age

**Figure 2.**
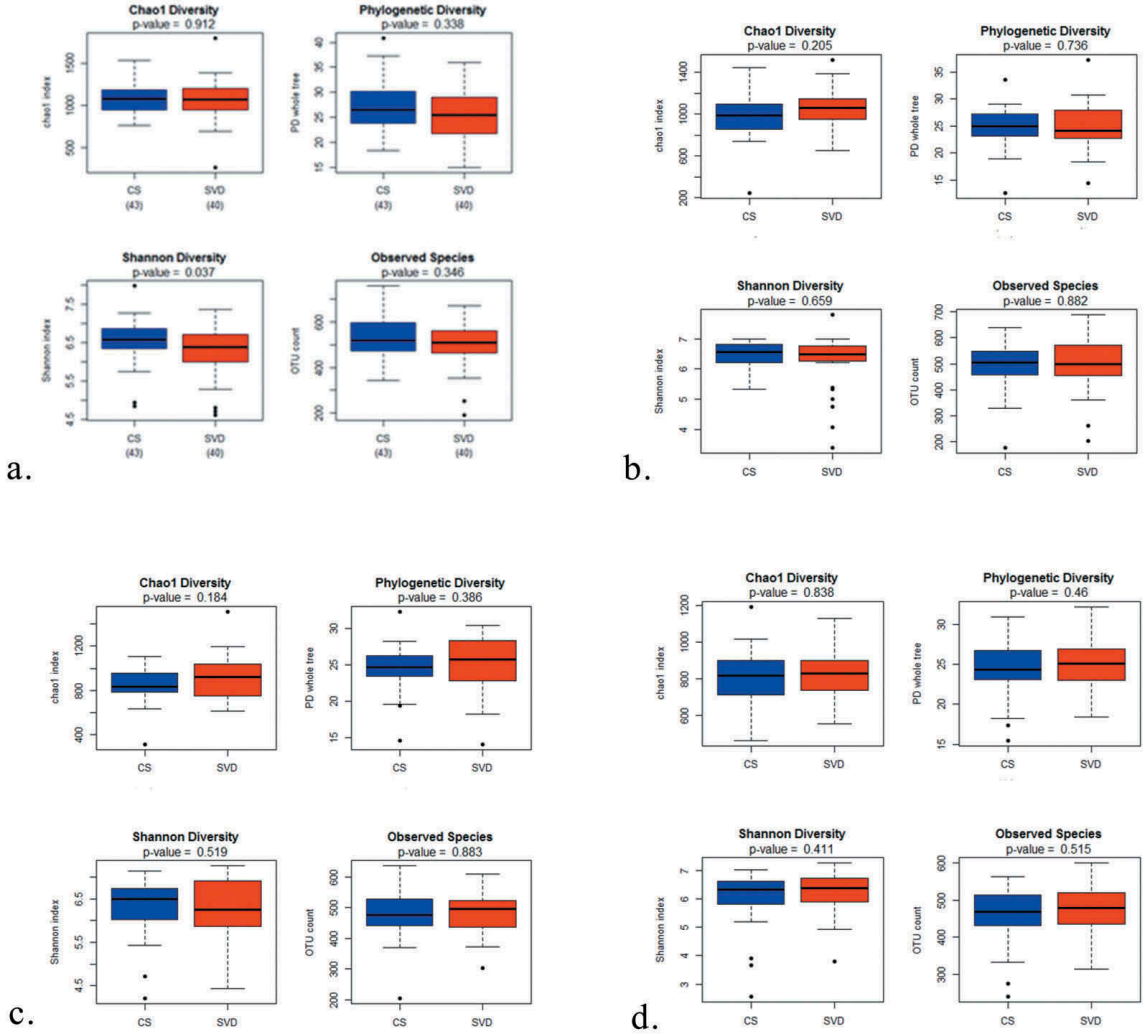
Alpha-diversity measurement of the influence of mode of delivery (SVD vs. CS) on the infant oral microbiota at various time points (week 1, week 4, 6 months and 1 year). Boxplot of the chao1 diversity, observed species, phylogenetic diversity and Shannon diversity in the two groups (SVD and CS) at (a) week 1, (b) week 4, (c) 6 months and (d) 1 year. Outliers are represented by black points.

To investigate the influence of birth mode on the oral microbiota composition as the infant increases in age, infant oral microbiota data were separated based on birth mode (SVD or CS) at the various time points (week 1, week 4, 6 months and 1 year). Alpha diversity was used to measure the overall diversity of the community present in the sample. Four indices were used (see Figure 2). The alpha diversity as represented by Shannon diversity index, of the infant oral microbiota at 1 week of age, was influenced by birth modality (*p*-value < 0.037), but at an older age, there was no influence of birth mode on the oral microbiota of the infant. Shannon diversity index takes the abundance of species into account, and in this case, this index indicates that the diversity of the infant oral microbiota at week 1 is lower in SVD infants, compared to CS infants. Therefore, the species abundance is richer in CS infant oral microbiota.

### The oral microbiota of the infant is influenced by delivery mode at 1 week of age, but not at an older age

**Figure 3.**
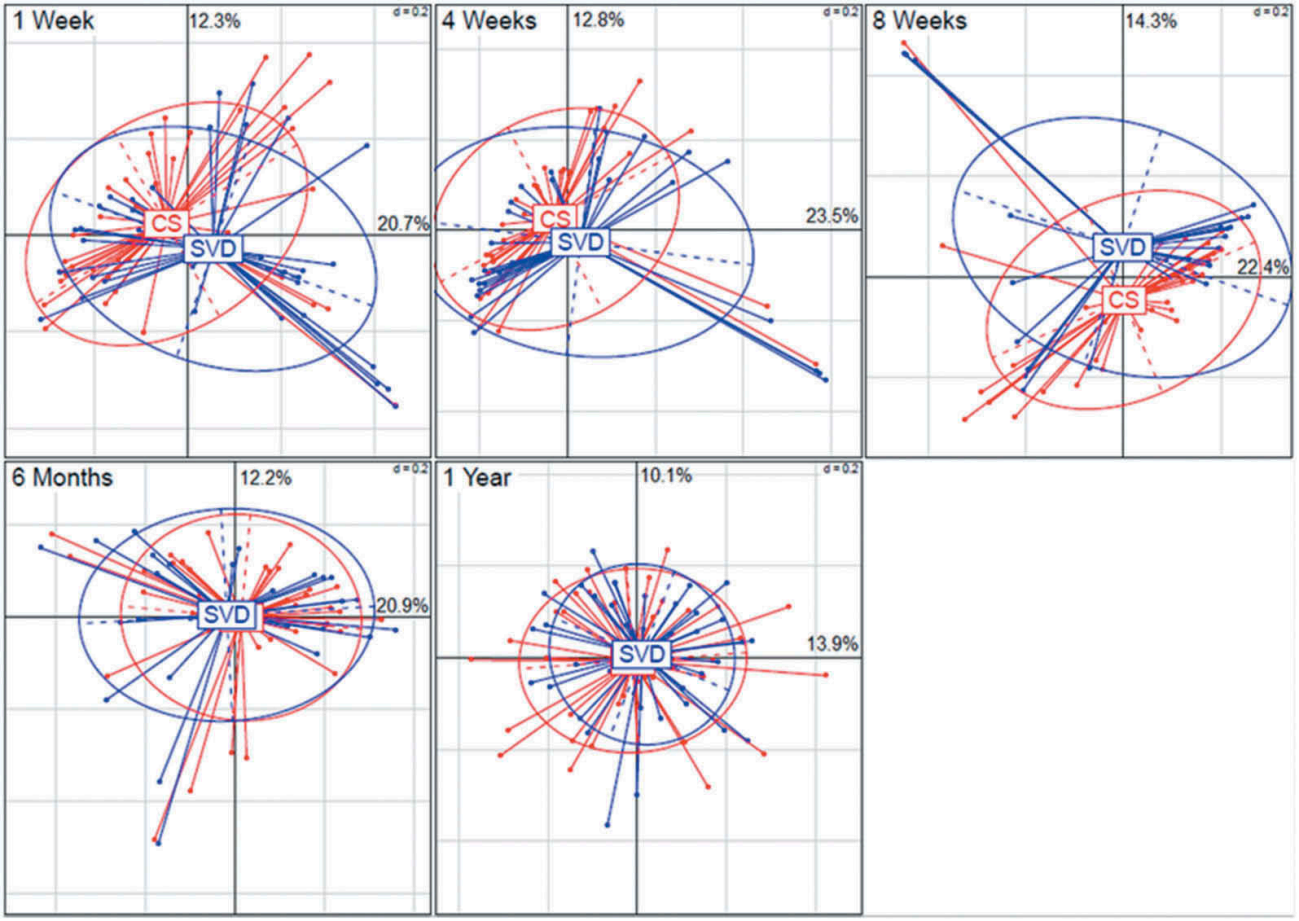
PCoA (principle coordinates) plots, each demonstrating the effect of birth mode (SVD vs. CS) on the oral microbiota composition of the infant at different ages (weeks 1, 4, 8, 6 months and 1 year). Significance was calculated using permutational multivariate analysis of variance (PerMANOVA) (Table 2).

We investigated the influence of birth mode (SVD vs. CS) on the oral microbiota composition of the newborn infant and significance was calculated using permutational multivariate analysis of variance (PerMANOVA) (Table 2). The results were presented by PCoA (principle coordinates) plots (see Figure 3). The beta diversity of the infant oral microbiota at 1 week of age was shown to be affected by birth mode (SVD or CS). The impact of birth mode on the infant oral microbiota at 1 week of age was statistically significant (*p* < 0.05). With increased age of the infant, we did not find any significant impact of birth mode on the infant oral microbiota composition (Table 2). Visually, the overlap/clustering between SVD and CS becomes tighter with older age, with nearly complete overlap by 6 months and 1 year, illustrating homogeneity of the oral microbiota within the oral cavity by birth modes.

**Table 2.** Significance values for the difference between SVD and CS at each time point.

Time point	*N* (number of infants) SVD	*N* (number of infants) CS	PerMANOVA *p*-value
1 week	34	41	0.047*
4 weeks	27	29	0.31
8 weeks	24	29	0.1
6 months	22	31	0.324
1 year	25	30	0.329

Significance was calculated using permutational multivariate analysis of variance (PerMANOVA). *p < 0.05; **p < 0.01; and ***p < 0.001.

#### The diversity of the oral microbiota up to 6 months of age remains stable but changes significantly from 6 months to 1 year of age

**Figure 4.**
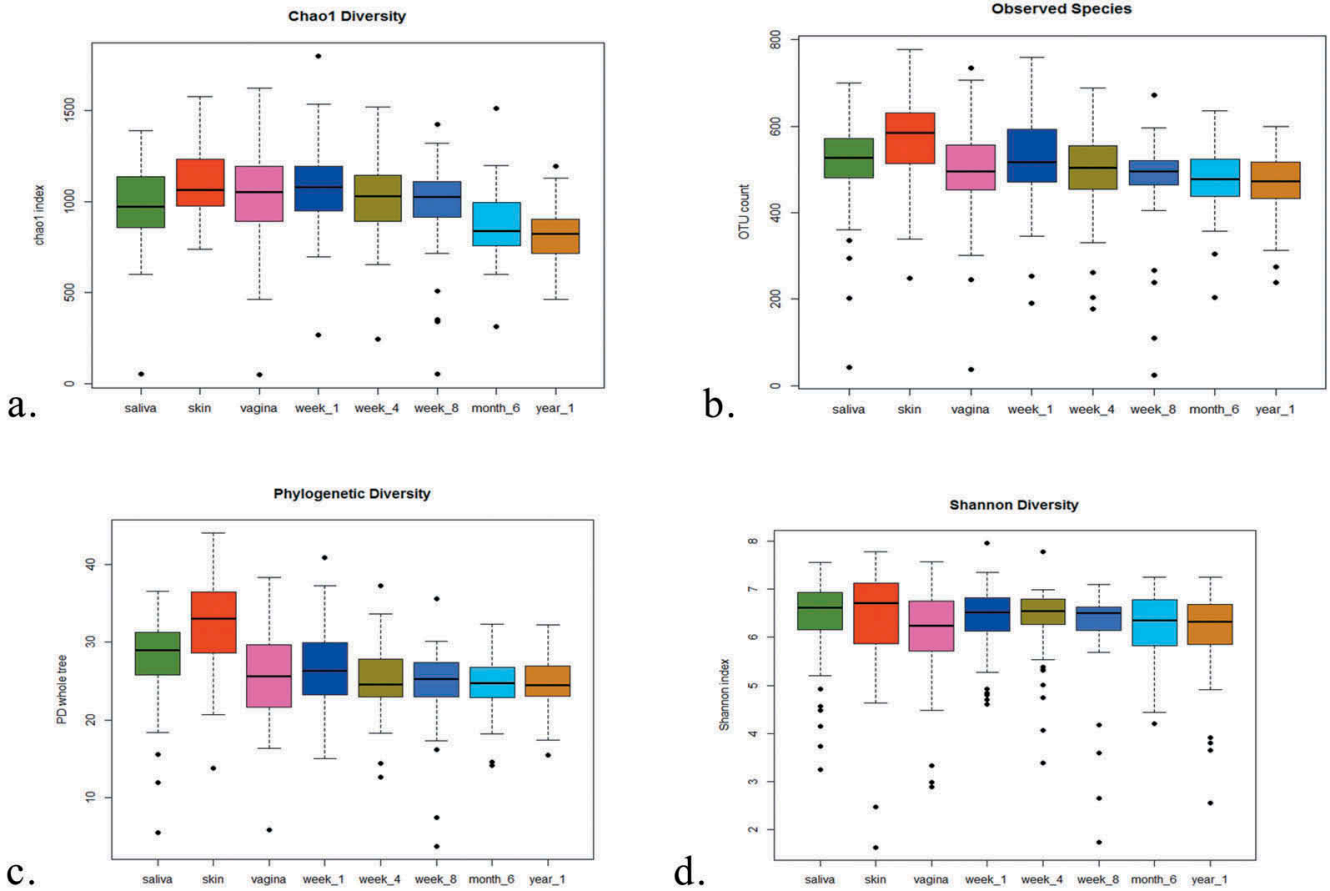
Alpha-diversity comparisons of the eight subject groups [(1) mother saliva, (2) Skin (mother) and (3) vagina (mother) and (4–8) infant oral weeks 1, 4, 8, 6 months and 1 year]. (a) Boxplot of chao1 diversity in the three groups, (b) boxplot of observed species in the eight groups, (c) boxplot of PD in the eight groups and (d) boxplot of Shannon diversity in the eight groups. Outliers are represented by black points.

To study the diversity of the microbiota from all samples, we calculated a series of alpha-diversity metrics: the Chao index, phylogenetic diversity (PD whole tree), observed species (OTU count), the Simpson index and the Shannon index (see Figure 4 and Supplementary Material 2). Alpha diversity is a measure of the diversity within a sample. The Chao diversity (see Figure 4(a)) which calculates species diversity within the sample and observed species (OTU count) index, which measures OTUs observed in a sample (Figure 4(b)), both illustrated that at the alpha diversity of the year 1, the oral microbiota was lowest and the alpha diversity of the oral microbiota decreased steadily over time from week 1 to year 1. However, the two remaining indices measuring alpha diversity, PD (see Figure 4(c)) and Shannon diversity (see Figure 4(d)), demonstrate that the diversity of the oral microbiota remains stable with increasing age.

All metrics illustrated that of the samples analysed, the skin microbiota diversity was highest, with high abundance of *Propionibacterium, Streptococcus*, and *Corneybacterium*, and the diversity of the vaginal microbiota was lower than the skin microbiota, composed mainly of genera *Streptococcus, Haemophilus, Neisseria* and *Lactobacillus*, while both the maternal skin and the vaginal microbiota demonstrate compositional differences (*p* < 0.001).

When species diversity was measured by Chao diversity, we observed that the maternal oral microbiota and the infant oral microbiota at week 1 of age are different (*p* < 0.005), while at 1 year of age, the alpha-diversity difference between these two samples is still of significance (see Figure 3(a)). By the other alpha-diversity measures, the PD measurement illustrated similar findings at week 1 (*p* < 0.05), while the OTU count showed difference in diversity of significance from 8 weeks (*p* < 0.05). These results demonstrate that at a younger age, the differences in microbiota diversity between mother saliva and infant saliva are less than at an older age, highlighting some relatedness at an early age between the oral microbiota of mother and infant.

The composition of the oral microbiota of the infant does not appear to change between 1 and 8 weeks of age when measured using Chao diversity and Shannon diversity. As measured by the Shannon index (see Figure 3(d)), oral microbiota diversity differences are apparent from 6 months of age, with oral microbiota differences evident between 6 months and 1, 4 and 8 weeks (*p* < 0.0001) and 6 months and 1 year (*p* < 0.05). These metrics demonstrate that the diversity of the oral microbiota remains relatively stable from 1 to 8 weeks of age and it is not until the infant reaches 6 months of age and 1 year of age do we see significant changes in the diversity of the infant oral microbiota.

### The infant oral microbiota does not demonstrate clear clustering by differential taxon abundance from week 1 to 1 year of age

In this analysis, we compared the abundance levels of the taxa in the maternal (skin/vagina/saliva) data sets along with the infant oral microbiota data sets at the various ages. In Figure 7, as illustrated by hierarchical clustering, we compared the microbiota data sets from mother (saliva) and infant oral microbiota pairs, at genus level, and clustering of mother–infant pairs was presented on a cluster dendrogram (see Supplementary Figure 1).

**Figure 5.**
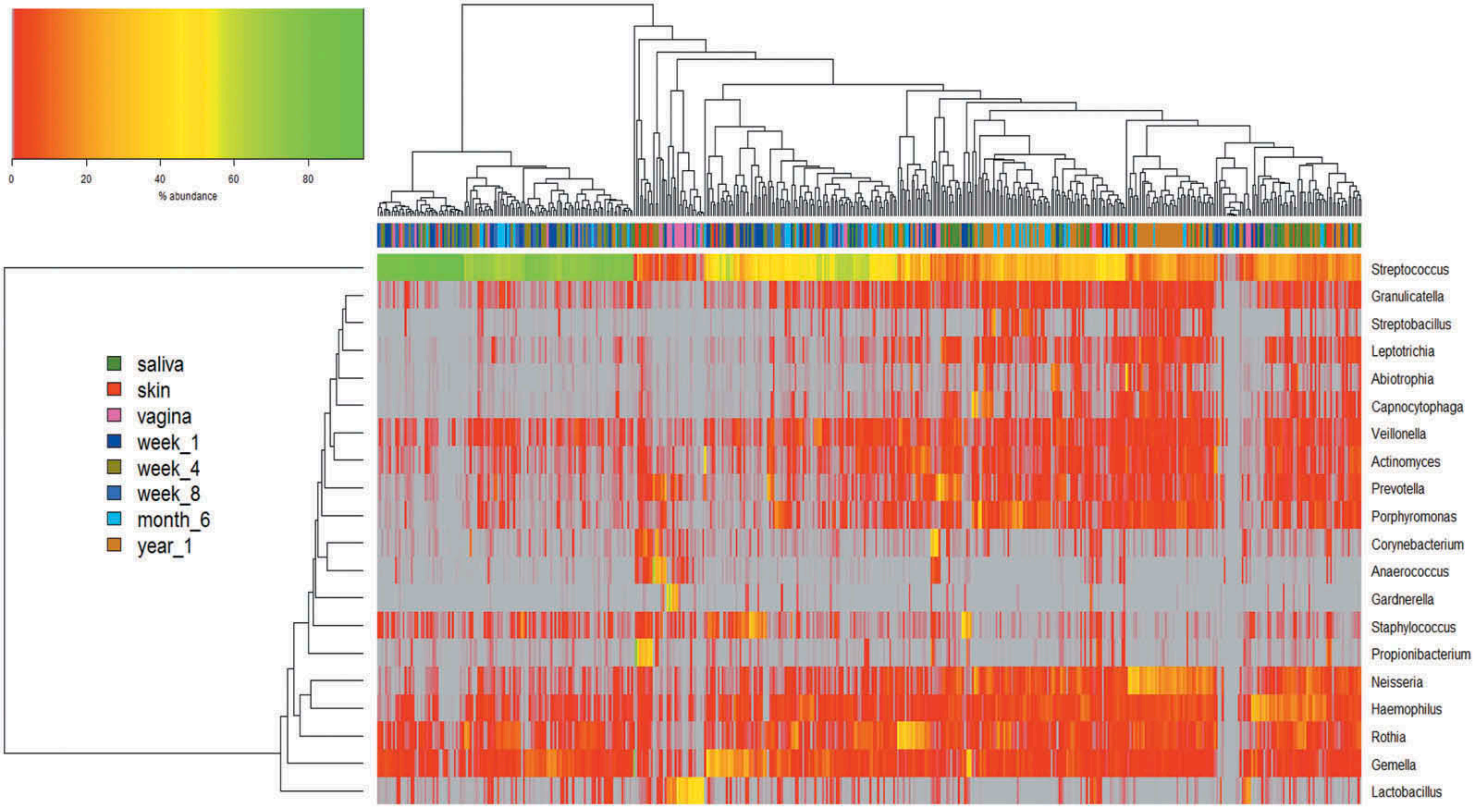
Hierarchical clustering of microbiota data at genus level. Abundances are colour-coded according to the colour key on the top left with grey representing a value of zero. Euclidean distance and complete linkage were used to cluster the rows and columns of the heatmap. The colour bar on side of the heatmap corresponds to sample type. Each genus with a mean ≥0.5% across all samples was included. All taxa present at less than 1% in all groups are excluded from the heatmap.

The abundance of bacterial taxa at genus level, of the maternal (skin/vagina/saliva) data sets along with the infant oral microbiota data sets at the various ages, is illustrated in Figure 5. There is evidence of two main branches in the horizontal dendrogram above the colour bar. The first branch illustrates clustering of samples, mainly of oral origin from the infant at weeks 1, 4, 8 and 6 months, with a very high abundance of the genus *Streptococcus* (see Figure 5). Interestingly, the year 1 oral samples from the infant appear clustered to the right, together with some 6 months infant oral samples, with 6 months samples seen as a transition between the early infant oral microbiota (weeks 1–8) to later ages of 1 year. The genera that are abundant in the year 1 infant oral microbiota include *Neisseria, Haemophilius, Porphyromonas* and *Streptococcus*. At 1 year of age, there are more diverse genera present, compared to 1 week of age of the infant, where the diversity is less, and only a few genera dominate, such as *Streptococcus*. Although we do not see a distinct trend where the oral microbiota cluster based on increased age of the infant, we do however see that at the early ages of the infant, weeks 1–8, there is tighter clustering, compared to the later stages where 6 months are too closer to 1 year of age. This trend is supported by our alpha-diversity measures (see Figure 2), where from 6 months, the difference in diversity becomes more significant than the earlier time points.

The maternal saliva microbiota data appear to be distributed throughout the horizontal dendrogram, with small areas of tight clustering, highlighting its high diversity, with increased abundances of genera *Streptococcus, Neisseria, Haemophilus* and *Prevotella*. There are high abundances of genus *Streptococcus* numbers in both samples, with the genus *Lactobacillus* in very high abundances in the vaginal samples only. The genera associated with the skin samples are more variable, with higher diversity, including *Propionibacterium, Haemophilus* and *Staphylococcus*.

**Figure 6.**
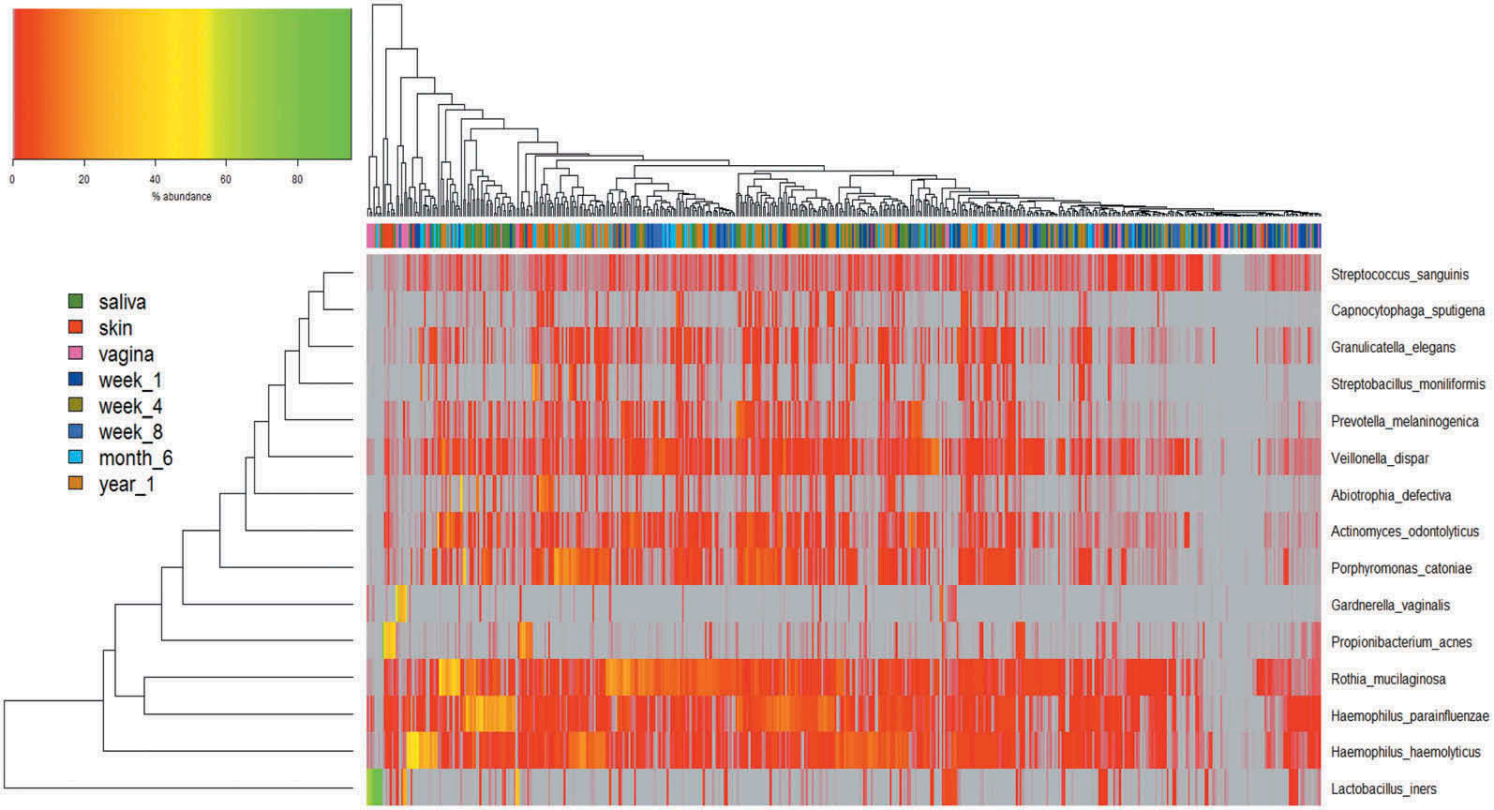
Hierarchical clustering of microbiota data at species level.

We included in this analysis all samples with at least one species present with a median value of ≥0.5% across all samples, and this is presented in Figure 6. There was no obvious clear separation between each group of samples. We did however notice tight clustering of some samples, such as vaginal samples with high abundances of *Lactobacillus iners*, skin samples with high abundance of *Propionibacterium acnes* and saliva samples with high abundances of *Rothia mucilaginosa, Haemophilius parainfluenzae* and *Haemophilius haemolyticus*.

Abundances are colour-coded according to the colour key on the top left with grey representing a value of zero. Euclidean distance and complete linkage were used to cluster the rows and columns of the heatmap. The colour bar on top of the heatmap is coloured according to sample type. All taxa present with at least one species with a median value of ≥0.5% across all samples are included.

**Figure 7.**
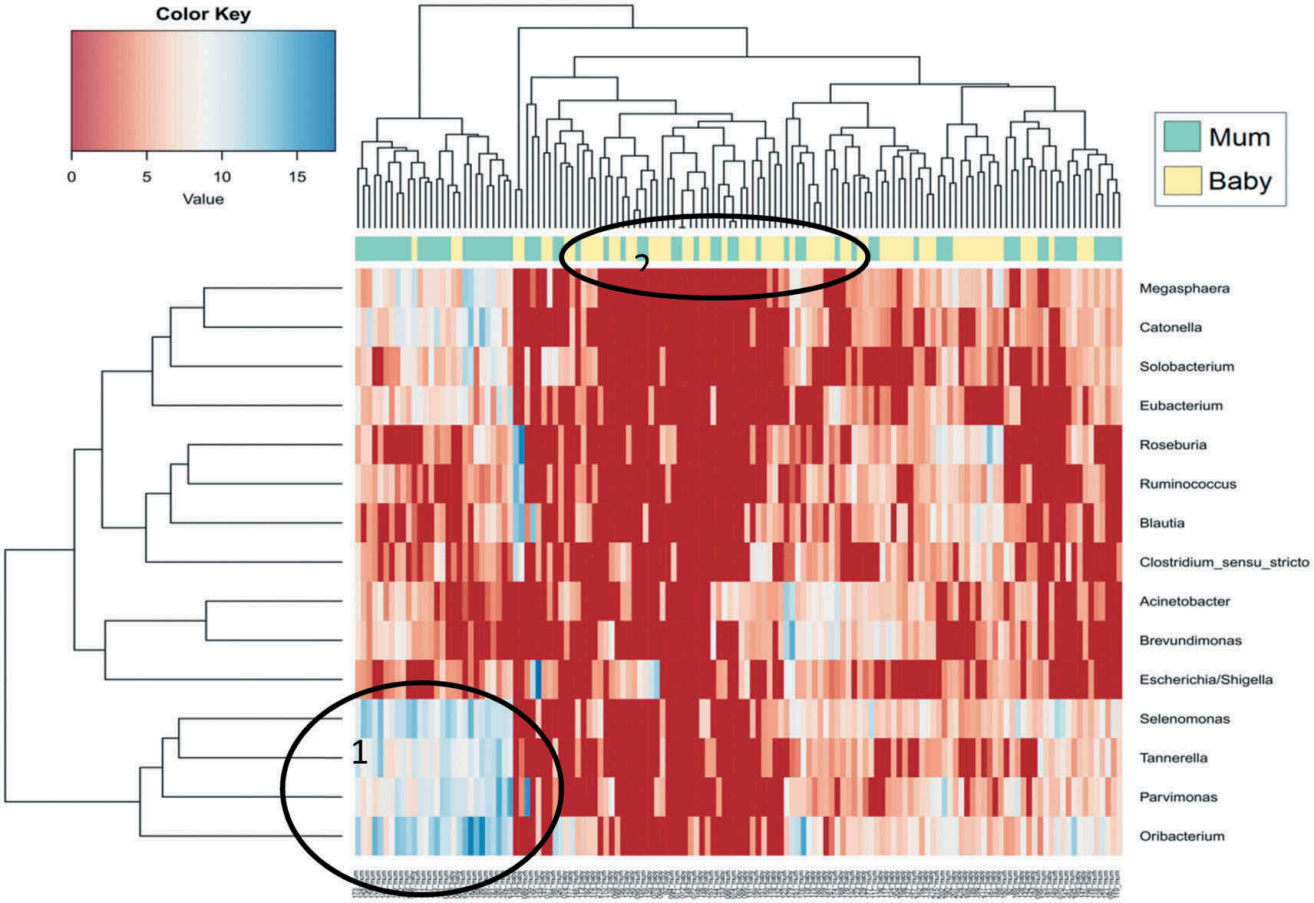
Hierarchical clustering of data between maternal saliva microbiota and infant oral microbiota at bacterial genus level. Abundances are colour-coded according to the colour key on the top left with red representing a value of zero. Euclidean distance and complete linkage were used to cluster the rows and columns of the heatmap. The colour bar on side of the heatmap corresponds to sample type (green = mother, yellow = infant). All taxa present at less than 1% in all three groups are excluded from the heatmap.

We compared the saliva microbiota of the mother with the infant oral microbiota and bacterial taxon abundance was illustrated on a vertical and horizontal dendrogram (see Figure 7). This analysis did not identify individual mother-to-infant pairs clustering together (see Supplementary Figure 1) but did identify clustering of maternal saliva microbiota and infant oral microbiota as separate groups. One subset of mothers illustrated a high abundance of the genera *Selenomonas, Oribacterium* and *Tannerella* (see Figure 7: labelled 1), and a lower abundance of genera, such as *Ruminococcus* and *Escherichia*/*Shigella*. Infant sample clustering was evident, with genera, such as *Selenomonas, Oribacterium* and *Megasphaera*, in higher abundance (see Figure 7: labelled 2).

### Compositional differences at phylum, genus and species level distinguish maternal and infant microbiota data sets

**Figure 8.**
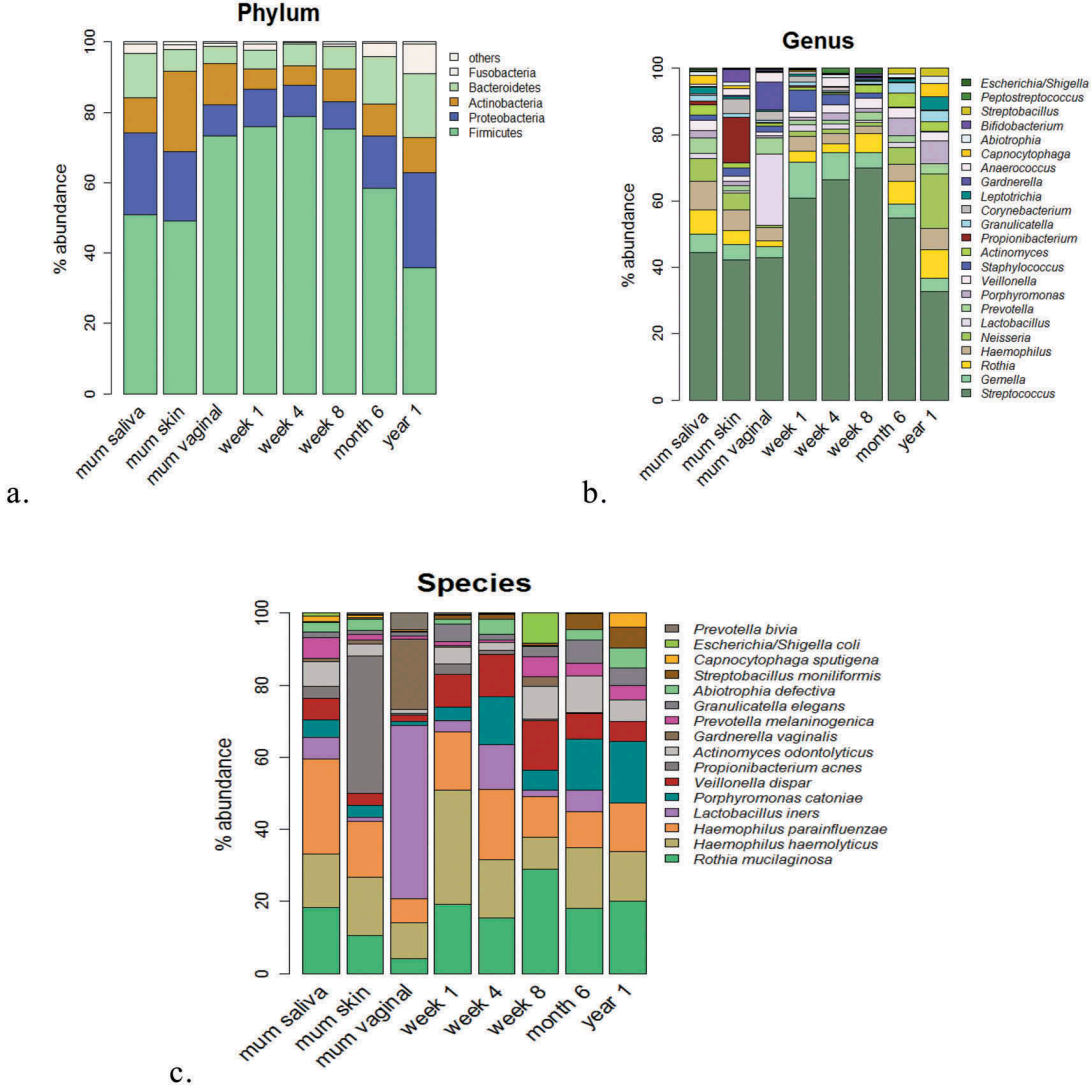
Broad and fine detail compositional differences at genus, phylum and species level. (a) Microbiota composition at phylum level. Percentages for each taxon represent the median abundance values for the sample types. (b) Bar plot of percentage abundance at the genus level. Percentages for each taxon represent the median values for the groups. (c) Bar plot of percentage abundance at species level. Percentages for each taxon represent the median values for the groups.

We assessed all samples in each group (mother: skin, vagina and oral/saliva; infant oral at weeks 1, 4, 8, 6 months and 1 year) for their compositional differences at phylum, genus and species level (see Supplementary Material 1). The relative abundances of each group are presented at each level (see Figure 8). The mothers’ oral saliva is dominated by *Firmicutes*, followed by *Proteobacteria* and *Bacteroidetes*. The skin and vagina both have a mixed composition also dominated by *Firmicutes*, while the mothers skin has increased *Proteobacteria* and *Actinobacteria*, and both body sites are statistically different in their microbiota composition (*p* < 0.001) (see Figure 8(a)).

At phylum level (see Figure 8(a)), the composition of the oral microbiota between week 1 and 1 year is different (*p* < 0.001), while only from 6 months do we see the significance begin to emerge. The infant oral saliva microbiota is dominated from birth to 6 months by *Firmicutes* and is relatively stable in its abundance from week 1 to 6 months (see Figure 8(a)). *Firmicutes* levels between week 1 and week 8 are similar with relative abundances at week 1 (82.3%), week 4 (87.3%) and 8 weeks (81.1%). Only after 8 weeks does the abundance of *Firmicutes* begin to decrease, from 6 months (60.2%) until 1 year (33.7%), and this coincides with an increase in the abundances of *Proteobacteria* at 1 year (25.6%) (*p* < 0.0001) and *Bacteroidetes* (16.7%). These changes from 6 months to 1 year may be due to the influence of tooth eruption and introduction of solid foods, which typically occurs at this time.

As expected, at genus level, the dominant genera of the infant oral microbiota at 1 week of age is *Streptococcus*, with *Streptococcus* levels gradually increasing in abundance, from week 1 (53.8%) to week 4 (67.6%) and week 8 (67.5%) (see Figure 8(b)). However, beyond 8 weeks, this trend ceases, with decreasing levels of *Streptococcus* at 6 months (42.8%) and again at 1 year of age (23.1%). When *Streptococcus* levels begin to decrease, this is counteracted by increased abundances by year 1 of genera, such as *Neisseria* (10.3%), *Porphyromonas* (3.96%), *Rothia* (3.68%) and *Haemophillus* (3.87%).

By 1 year of age, the infant microbiota is different to the microbiota at a younger age (*p* < 0.001), and there is increased presence of genera, such as *Streptococcus, Porphyromonas, Neisseria* and *Haemophilus*. Although the microbial composition within the oral microbiota of an infant aged year 1 is composed of genera similar to the mothers’ microbiota, their microbial composition is different (*p* < 0.001) (see Figure 8). From week 1 to year 1, species that dominate the oral microbiota include *R. mucilaginosa, H. haemolyticus, H. parainfluenzae, Porphyromonas catoniae, Veillonella dispar, L. iners* and *Prevotella melaninogenica*.

**Figure 9.**
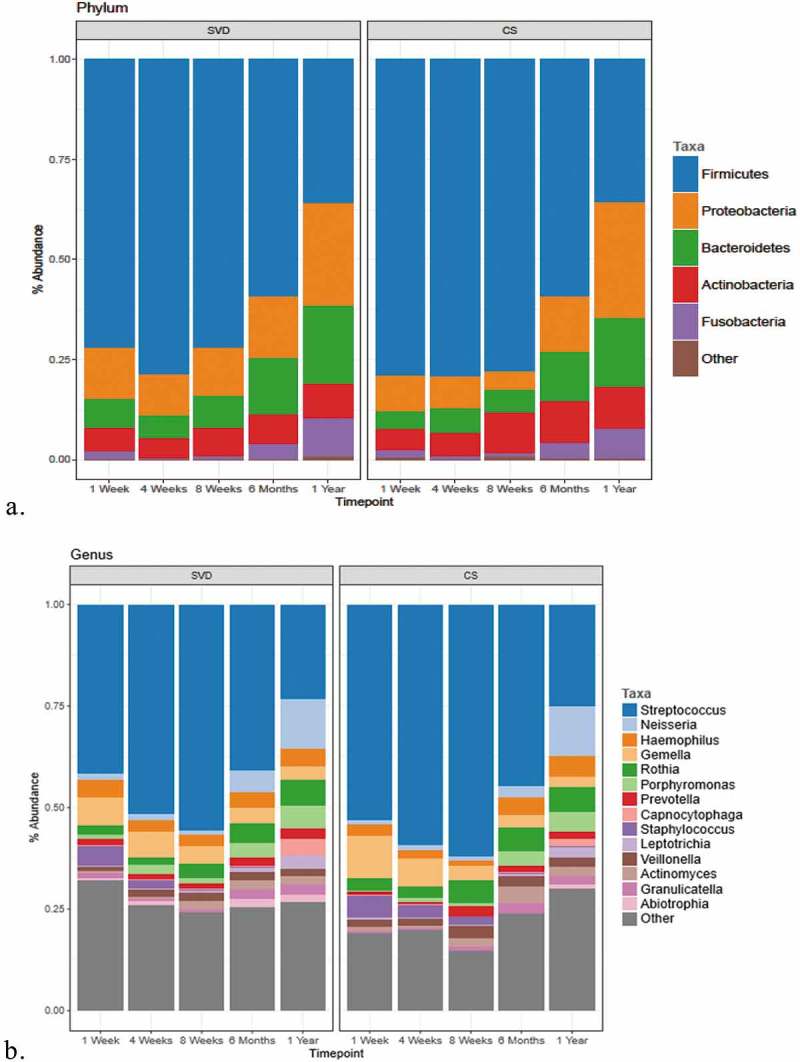
Comparison of the microbiota composition of infants born by different birth modes (SVD and CS) across five time points from 1 week to 1 year of age. (a) Microbiota composition at the phylum level. Percentages for each taxon represent the median abundance values for the sample types. (b) Bar plot of percentage abundance at the genus level. Percentages for each taxon represent the median values for the groups. The graph includes data for genera found at >1% average in the total population. Genera found at <1% were grouped as ‘other’.

The bacterial composition of the mothers’ skin includes *Propionibacterium, Streptococcus* and *Corneybacterium*, and the species present include *P. acnes, H. parainfluenzae*/*haemolyticus* and *R. mucilaginosa*. The vaginal microbiota composition is made up mainly of the genera *Streptococcus, Haemophilus, Neisseria* and *Lactobacillus*, and species *L. iners* is the main *Lactobacillus* species identified in the vaginal microbiota of the mother and makes up the majority of the composition, followed by *Gardnerella vaginalis* (see Figure 8(c)). The influence of birth mode on the oral microbiota from these maternal body sites (skin/vagina) is evident within 1 week of age only but not beyond (see Figure 2). *L. iners* is present at week 1 and surprisingly increases in abundances by week 4 in the infants’ oral microbiota, highlighting the potential transfer from mother (vaginal) to infant, along with the species *P. acnes* from maternal skin. To assess the effect of birth mode on the oral microbiota composition of the infant, we divided the oral microbiota of infants by birth mode (see Figure 9). At phylum level, for both CS and SVD delivered infants, there is a similar pattern in the phylum composition over time (see Figure 9(a)), while at genus level, at week 1, infants born by SVD appear to have lower levels of *Streptococcus, Gemella* and more *Porphyromonas* and *Prevotella* than CS infants at week 1.

### Breastfeeding duration does not influence the oral microbiota of the infant

**Figure 10.**
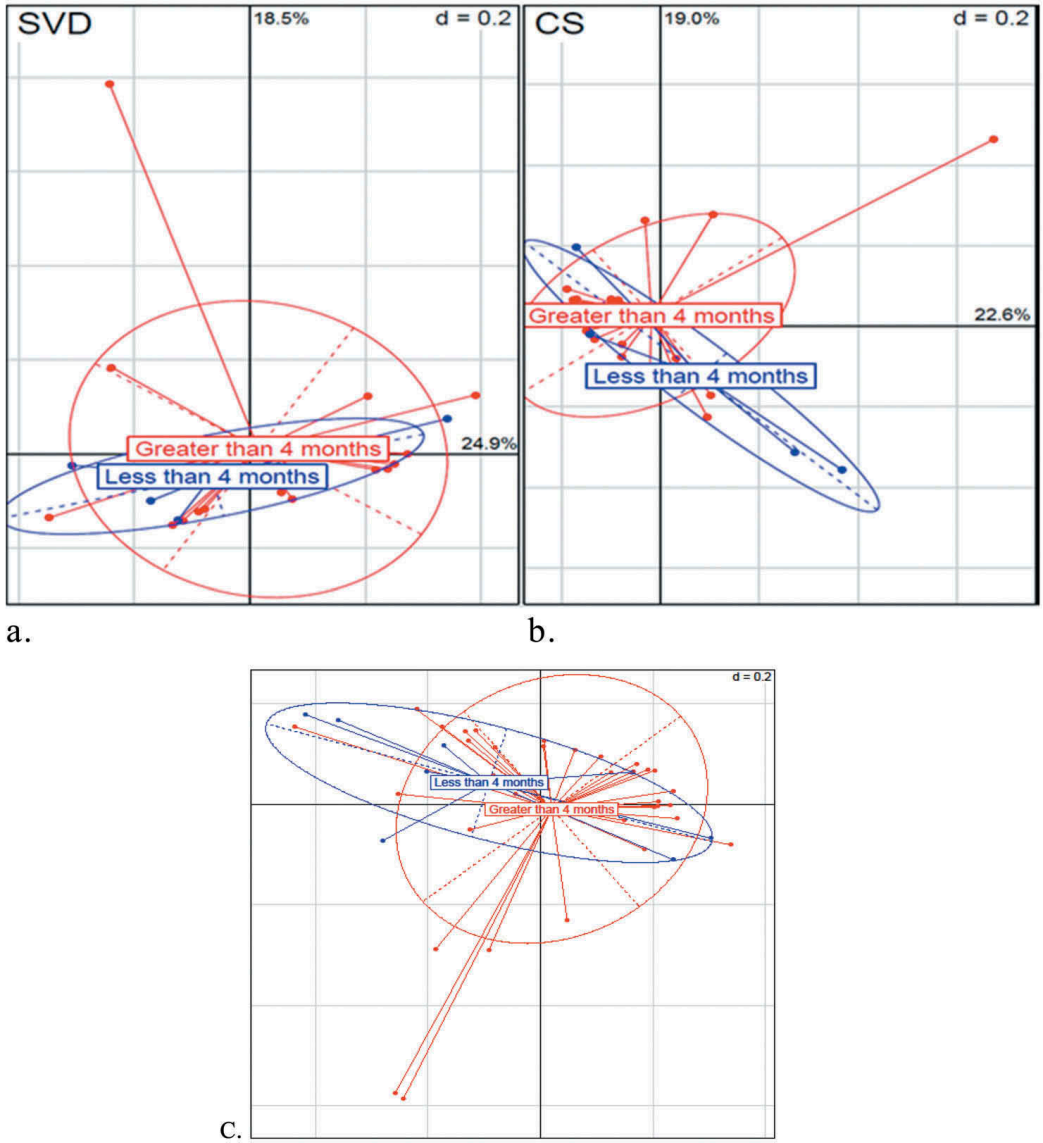
Beta-diversity PCoA (principle coordinates) plots (Bray–Curtis) illustrating the influence of breastfeeding duration on the oral microbiota of SVD and CS infants. (a) Naturally delivered infants (SVD). (b) CS infants. In blue are infants that were breastfed for less than 4 months. In red are infants that were breastfed exclusively for longer than 4 months. (c) Beta-diversity PCoA (principle coordinates) plots (Bray–Curtis) illustrating influence of breastfeeding duration on the oral microbiota of combined (CS and SVD) infants.

Breastfeeding duration data were collected for each infant from birth until 1 year of age including duration of exclusive breastfeeding. Two categories were recorded: less than 4 months and greater than 4 months duration of breastfeeding (Table 3), as referenced by Hill et al. [5]. Significance of microbiota composition comparison was calculated using PerMANOVA (Table 4) and presented by PCoA (principle coordinates) plots (see Figure 10). No differences in the oral microbiota of the infants were detected when separated by birth mode (SVD and CS) and the duration of breastfeeding (see Figure 10). When infant microbiota data of both birth modes were combined, the duration of breastfeeding did not influence the infant oral microbiota (see Figure 10(c)). Thus, this analysis indicates that breastfeeding, less than or greater than 4 months, does not influence the oral microbiota of infants, independent of birth mode.

**Table 3.** Total sample number of exclusively breastfed infants included in the analysis whom breastfed for greater or less than 4 months.

Birth mode	*N* (number of infants)	Breastfeeding duration	
		>4 months	<4 months
CS	22	18	4
SVD	22	18	4

CS: Caesarean section; SVD: spontaneous vaginal delivery.

**Table 4.** Significance values (*p*-values) calculated using permutational multivariate analysis of variance (PerMANOVA) for the difference between ‘less than 4 months’ breastfeeding’ and ‘greater than 4 months’ breastfeeding’ for SVD and CS infants.

Birth mode	*N* (number of infants)	PerMANOVA (*p*-value)
CS	22	0.246
SVD	22	0.359
Combined (SVD and CS)	44	0.23

CS: Caesarean section; SVD: spontaneous vaginal delivery. Both groups (CS and SVD) were combined and tested against duration of breastfeeding.

## Discussion

Our findings add to previous limited research focusing on birth mode influence on the infant oral microbiome and demonstrate that external influences, such as diet, siblings, environment, teething and ‘chewing’, for example toys, have more influence on the oral microbiota than birth mode, which influences the oral microbiota immediately after birth only. At birth, we found that the mother’s skin, vagina and oral microbiota communities are distinct, and for each body habitat (vagina, skin, oral cavity), they demonstrate a unique composition (see Figure 1). The diversity of the vaginal microbiota was lower than the skin microbiota of the mother as measured by alpha-diversity metrics (see Figure 4), similar to previous observations [20,54]. This low diversity within the vaginal samples is most likely due to the dominance of genus *Lactobacillus* with *L. iners* and *G. vaginalis* in high abundances, as represented by community state types (CST) III and CST IV [55]. These species have been previously identified in the vaginal microbiota of healthy women of the reproductive age [56–58] and of pregnant women [55] particularly in the last trimester [59].

All metrics illustrated that of the samples analysed, the skin microbiota diversity was highest, with high abundance of *Propionibacterium, Streptococcus* and *Corneybacterium*, and the diversity of the vaginal microbiota was lower than the skin microbiota, composed mainly of the genera *Streptococcus, Haemophilus, Neisseria* and *Lactobacillus*, while both the maternal skin and vaginal microbiota demonstrate compositional differences (*p* < 0.001). The diversity of the skin is high due to its open and exposed environment although the diversity can vary by the sample site location [60–62], while the vaginal microbiota can be dominated by fewer species such as *Lactobacillus*.' The vaginal microbiota diversity was previously found to be the lowest compared to skin and oral cavity of adults sampled [20,54].

In agreement with previous studies [15,43], we found that the birth mode had an influence on the neonatal oral microbiota for a short window after birth. We detected that the oral microbiome of the infant was influenced by birth mode within 1 week of age only, but from 4 weeks of age to 1 year, the effect of birth mode was not apparent. The overlap and tight clustering presented by PCoA plots (see Figure 3) of the infant oral microbiota by birth mode are obvious by 6 months and 1 year of age, while the beta diversity between samples (see Figure 1) demonstrates sharing of taxa between the maternal skin and vaginal microbiota and the oral microbiota of the infant at weeks 1, 4 and week, with less overlap and less effect of the maternal microbiota with increased age (6 months and 1 year). This early stage impact highlights transfer of maternal microbiota, respective to their birth mode (CS or SVD) to the infant, further reinforced by the identification of *L. iners* and *P. acnes* of vaginal and skin origin in the infants’ oral microbiota within 1 week of birth. While the impact of the maternal vagina and skin are apparent, the maternal salivary oral microbiota does not have any significant effect on the infant oral microbiota, with no clustering of mother–infant pairs when compared by their oral microbiota composition only (see Figure 8) (see Supplementary Material 2). We identified that there was no impact of the maternal oral microbiota on their own infant oral microbial community, as demonstrated by Dominguez-Bello et al. [15], and we also found no direct similarity between the mothers’ salivary microbiota and their infants’ salivary microbiota, consistent with previous findings [42,43], reflecting compositional differences between the edentulous neonate and the established and mature adult oral microbiome.

Studies have reported increased diversity associated with SVD delivered infants [16,17]. We did not identify such diversity difference by alpha-diversity measures. Birth mode and age have both been shown to affect the microbial composition of the infant gut microbiota up to 6 months [5]. This influence was not seen for the same duration in the oral microbiota of the infants beyond 4 weeks of age (Figure 4). This supports previous research findings that as the infant ages, the influence of mode of delivery on the oral microbiota of the infant lessens [17]. We did however demonstrate that the diversity of microbes in the oral cavity of the infant decreased slightly with increased age as measured by Chao diversity and Shannon diversity, while stability within the oral cavity was demonstrated from week 1 to 1 year of age by the remaining alpha-diversity measures (phylogenetic and Shannon diversity) (see Figure 4). While one would expect to see increasing diversity with increased age, we found the alpha diversity to be relatively stable over time, perhaps symbolising low inter-variation differences between the oral microbiota of the infant over time. While the diversity did not dramatically change from 1 week to 8 weeks and again to 1 year, there were significant compositional changes which signify the role of factors, such as diet, dentition, salivary flow rate, oral health, siblings etc., have to play. We did however observe a lower diversity at all ages in the infant oral microbiota compared to the maternal ‘adult’ salivary microbiota, with similar compositional differences which have been identified previously in the salivary microbiome between adult and infant [64–66]. The diversity gap between the mother and infant oral microbiota increased as the infant grew in age demonstrating closer relatedness at a younger age. Both findings highlight that at 1 year of age, the diversity is stable, and not until an older age are diversity changes evident, potentially influenced by a myriad of factors (diet, oral health, dental factors, social status, salivary flow rate, fluoridation status and education).

The infant oral microbiota illustrates tight overlap by beta-diversity measures (see Figure 1) at 1 week of age to 8 weeks of age, but from 6 months of age, the infant oral microbiota develops its own distinct makeup with a gradual transitioning away from this early age taxa (week 1–8), with no overlap or clustering by 1 year of age as presented on the PCoA plots (see Figure 1). This highlights the continued development and maturation of the infant oral microbiota with its distinct composition, with no influence of birth mode at this age, with studies illustrating that oral microbiota composition can continue to change and develop into adulthood [64]. At the phylum level, the infant oral microbiota of infants from week 1 to week 8 was dominated by *Firmicutes* with *Streptococcus* the predominant genus at week 1, consistent with previous studies of the oral microbiome of infants [42] and of children [67]. Only after 8 weeks does the abundance of *Firmicutes* begin to decrease, from 6 months (60.2%) until 1 year (33.7%), while *Streptococcus* levels gradually decrease in abundance from week 1 (53.8%) to 1 year of age (23.1%). When *Streptococcus* levels begin to decrease, this is counteracted by increased abundances by 1 year of age with genera, such as *Neisseria, Porphyromonas*, *Rothia, Gemella* and *Haemophillus*, highlighting compositional changes within the oral microbiota from the neonatal period into toddlerhood. These compositional changes, in particular, are most apparent from 6 months of age, coinciding with the average eruption of the first deciduous teeth typically around the average age of 4–6 months for lower primary incisors and introduction of solid foods to the infant, where studies have seen changes in the oral microbial composition with eruption of teeth [68] and in the infant gut microbiota after introduction of solid foods [13,69].

We are aware that there are limitations to this study which include sample collection of the infant at the various time points, not recording the exact eruption of deciduous dentition while sampling, and precision diet analysis specifically focusing on the breastfeeding duration, formula feeding frequency and recording the details of the diet at the weaning stage from 6 months. In summary, sampling of these infants at each time point after birth was undertaken in their own home, and unfortunately due to personnel constraints, we were unable to do a dental check at each time point until 1 year, to observe the exact timing of eruption of the deciduous dentition, which would have benefitted this study. While the exact timing of teeth eruption was not recorded, antibiotic intake was recorded along with duration of feeding modality where all infants were breastfed for a minimum of 4 weeks. Long-term breastfeeding has been linked with increased risk of caries [70–72] and feeding modality (breastfed or formula-fed) has been shown to have an effect on the oral microbial composition of the infant [36,37]. We assessed if breastfeeding for a minimum of 4 weeks only had an effect on the oral microbiota of the infant, and we did not observe any significant effect of breastfeeding duration on the oral microbiota of infants born by SVD or CS, or when birth modality was combined (see Figure 10). Perhaps a longer duration of breastfeeding may have an impact on the oral microbiota, but all infants in this study were breastfed for a minimum of 4 weeks only, some with introduction of mixed-feeding (formula and breast) after this stage. These data were not collected in this cohort.

## Conclusion

This study indicates that the mode of delivery does not have any major influence on the infant oral microbiota after 4 weeks of birth. We observed the influence of birth mode on the oral microbiota of the infant by 1 week of age only. Changes in diversity and composition were observed in the oral microbiota of the infant over time. These changes are more visible at 6 months and beyond, and again at 1 year of age, when both teeth begin to emerge, and weaning of introduced food begins. Our findings provide a closer insight into the oral microbiota development from birth, and the influence of birth mode together with the documented changes in diversity and composition will aid us to get a better understanding of the long-term health impact within the oral cavity for the infant and provide a platform for additional studies to establish how early life disturbances can impact the oral health outcome of these infants.
